# Long-term outcome of interventional approaches for treatment of coronary artery fistulas: a retrospective cohort study in a great referral center

**DOI:** 10.1186/s43044-023-00339-4

**Published:** 2023-03-27

**Authors:** Avisa Tabib, Hojjat Mortezaeian, Mohammad Mahdavi, Yasaman Khalili, Nikolaus A. Haas, Sepideh Mohammadhoseini

**Affiliations:** 1 Heart Valve Disease Research Center, Rajaie Cardiovascular Medical and Research Center, Iran University of Medical Sciences, Tehran, Iran; 2 Rajaie Cardiovascular Medical and Research Center, Iran University of Medical Sciences, Tehran, Iran; 3 Department of Pediatric Cardiology and Pediatric Intensive Care University Hospital Munich LMU, Ludwing Maximilians University Marchioninstr, Munich, Germany

**Keywords:** Interventional approaches, Coronary artery fistulas, Patients

## Abstract

**Background:**

Despite the spontaneous regression of many cases of coronary artery fistulas (CAFs), symptomatic patients or cases with severe shunting may require therapeutic interventions. In the present study, we aimed to assess the outcome of treatment of CAFs using interventional approaches.

**Methods:**

This retrospective cohort study was performed on 29 patients with CAFs that were referred to our tertiary center between 2009 and 2019. Baseline characteristics were collected by reviewing the hospital files, and the patients were followed up to assess long-term outcomes with a mean follow-up of 3.3 years.

**Results:**

Overall, in 29 patients in our cohort study, 82.9% suffered from isolated CAFs and in the remaining cases, concurrent congenital abnormalities did exist. For treatment, coils (Cook, Pfm, Ev3) were used in 79.3%, ADO II(AGA) in 18.3%, vascular plug (AGA) in 3.4%, and a combination of coil/ vascular plug/amplatzer in 3.4%. Postoperative complications were reported in 4 patients as external iliac artery thrombosis, transient PSVT, ST-T wave changes and mild pericardial effusion that were all managed successfully with no adverse sequels. No coronary artery injury, device dislocation, dissection, ischemia or coronary dilatation occurred, and there was no death. As larger fistulas were treated by a retrograde approach through the right side of the heart, there was significant correlation between residual shunts and the mode of closure approach; the majority of the residual shunts occurred in patients in the retrograde approach group.

**Conclusions:**

Trans-catheter approach for treating CAFs leads to appropriate long-term outcome with minimal potential side effects.

## Background

Coronary artery fistula (CAF) is a rare disease and a component of congenital heart anomalies that involves a direct connection between the coronary artery system and one of the chambers of the heart, the coronary sinus or its associated veins, the pulmonary artery or pulmonary veins [1]. This phenomenon accounts for nearly 50% of congenital coronary anomalies and its diagnosis and treatment at an early age is important to prevent the progression of the disease [2]. In general, CAF is a rare vascular disorder and may be revealed in 0.05% of diagnostic catheterization cases [3]. Sakakibara et al. differentiate two types of CAFs: a proximal type, where the proximal of the CAF origin is dilated and the distal end is normal, and a distal type, where the CAF is dilated in its entire length [4]. Most of the CAFs originate from the left anterior descending artery (LAD) or right coronary artery (RCA) [5]. About 90% of CAFs drainage is to the right side of the heart [6], and left ventricular CAFs are much less common. The cause of CAFs may be congenital or acquired. Most cases of CAFs are congenital, but in some cases, CAF may be acquired due to chest trauma, myectomy, coronary angioplasty and bypass surgery [7]. Transthoracic echocardiography is very helpful and readily assessable in the diagnosis and follow-up of coronary fistula. Echocardiographic findings may vary according to the course of the fistula and may include abnormally dilated coronary arteries with tortuous course, enlarged cardiac chambers, dilated great veins, regurgitant flow via atrioventricular valves, diastolic flow reversal in aorta without aortic valve regurgitation, and reduced ventricular function [8, 9]. Because most patients are asymptomatic, many fistulas are found accidentally on coronary angiography or at autopsies [10]. Coronary angiography is the best way to prove the path and origin of these fistulas [11]. Clinical manifestations associated with CAFs depend on the type of fistula, shunt volume, shunt area, and other cardiovascular conditions [12]. Although the majority of patients are asymptomatic, functional shortness of breath, fatigue, pulmonary hypertension, congestive heart failure, bacterial endocarditis and dysrhythmia (especially atrial fibrillation) are the most common clinical manifestations in symptomatic patients [13]. In addition, this disease can have clinical symptoms at all ages. Last but not least, these clinical manifestations can range from a heart murmur to heart failure and myocardial infarction due to a steal phenomenon or embolization of a thrombus formed in the aneurysmal region [14]. Symptomatic patients with severe shunting may require surgical interventions for treatment, but today also percutaneous closure or coil embolization may be used for most patients who are candidate for surgery. Which method has the most favorable clinical outcome and lowest rate of side effects is still debated [15]. Extremely tortuous or multiple and diffused fistulas may not be suitable for catheter intervention, and in very small vessels in pediatric patient’s catheter intervention may not be a good choice [16]. Therefore, considering the importance of the issue, we aimed to investigate the outcome of the interventional treatment of coronary fistulas in our institution.

## Methods

This retrospective cohort study was performed on 29 patients with CAFs that were referred to our tertiary referral center between 2009 and 2019. Data collection included the hospital recorded files as well as angiographic information available in the hospital Picture Archiving Communication System (PACS). In this regard, along with baseline characteristics including demographics, the medical history, underlying disorders, diagnostic parameters, the anatomical location of the fistula and the trans-catheter method used, the interventional outcomes including recurrence of fistula, residual shunt, postintervention complications and death were assessed by reviewing the patients’ files. To assess the long-term outcome of the procedure, the patients were followed up for 3.3 ± 2.1 years (ranged 1 to 8 years), and in each follow-up, a physical examination, chest X-ray, electrocardiogram, transthoracic echocardiogram, and if it was necessary CT-angiography or catheter angiography was performed.

### Statistical analysis

For statistical analysis, the results are presented as mean ± standard deviation (SD) for quantitative variables and were summarized by frequency (percentage) for categorical variables. The categorical parameters were compared using the Chi-square test. *P* values of ≤ 0.05 were considered statistically significant. For the statistical analysis, the statistical software SPSS version 23.0 for windows (IBM, Armonk, New York) was used.

## Results

The baseline characteristics of the study subjects are summarized in Table 1. In total, 29 patients with CAFs who were treated interventionally were included in the study. The mean age of the patients was 44.0 ± 1.47 months (range 5 months to 14 years); 0.69% of patients were male and 31% were female. Overall, 82.9% suffered from isolated CAFs. In patients with fistulas and other abnormalities, these included a patent foramen ovale (PFO), single coronary ostium, atrial septal defect (ASD_2_), and previously total correction of Tetralogy of Fallot (TFTC) (17.1%). The fistula course in the patient with single coronary ostium was from RCA to RV, and the course of the acquired fistula in the Tetralogy of Fallot (TOF) patient previously undergoing TFTC was from the LMCA to RVOT. Overall, 58.6% of fistulas originated from the left coronary artery and the rest from the right coronary artery. Of the 29 patients studied, the most frequent fistula location was from the LMCA to RV, which was observed in 6 patients (20.7%), that was followed by a fistula course from RCA to RA in 5 patients (17.2%) and also RCA to RV in 5 patients (17.2%). There was one complex lesion that had LAD and RCA to RV fistula. Also, 93.1% of drainage location was to the right side of the heart (RA, RV, PA) and 3.4% (1 patient) drainage was to the left atrium and 3.4% (1 patient) drainage was to the coronary sinus. The most frequent clinical symptoms before interventional fistula treatment in patients were volume overload, which was observed in 21 patients (72.6%) followed by mild pulmonary hypertension (PH) in 17.2%, aneurysms of the LMCA in 6.8% and aneuryms of the RCA was detected in 3.4%, and there was no case of heart failure. Coils (COOK, PFM,EV3) were used for 23 patients (79.3%), while ADO II(AGA) was used for 4 patients (13.8%) and vascular plug(AGA) for 3.4%, and combination of coil/ vascular plug/amplatzer in 3.4%. A minimal residual shunt was observed in 24.2%, that all closed spontaneously. Also, 17.2% (5 patients) had a significant residual shunt after the initial procedure (RCA to RA, LCX to RV, LMCA to RV, LAD to RV, and LAD & RCA to RV shunts, each in one of the patients), which were closed successfully during a following intervention, and no case of CAFs recurrence was seen.

**Table 1:** Baseline characteristics of study population

Mean age, months	44.0 ± 1.47
*Gender, %*	
Male	20 (69.0%)
Female	9 (31.0%)
Mean weight, kg	16.4 ± 12.7
*Underlying defect*	
Isolated CAFs	24 (82.9%)
CAFs with patent foramen ovale	1 (3.4%)
CAFs with single coronary ostium	1 (3.4%)
CAFs with Tetralogy of Fallot total correction	1 (3.4%)
CAFs with small ASD_2_	2 (6.9%)

**Fig. 1:**
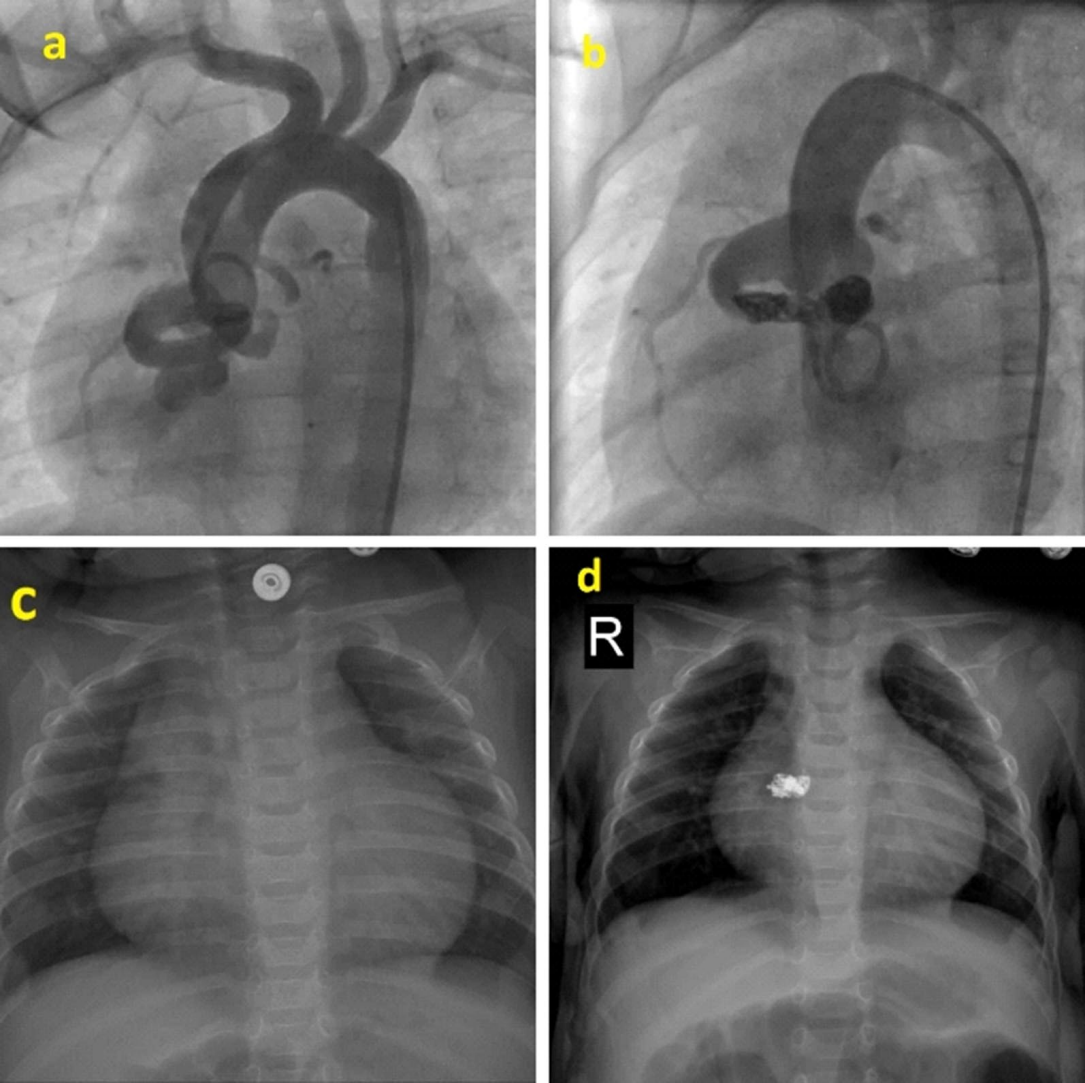
Fistula between RCA and RA which has been closed with 2 PFM coils via retrograde approach. **a**, **b**; Dilated fistula between RCA and RA, which has been closed with 2 pfm coils via retrograde approach. **c**, **d**; Reduced CT/ratio after the coil embolization

**Fig. 2:**
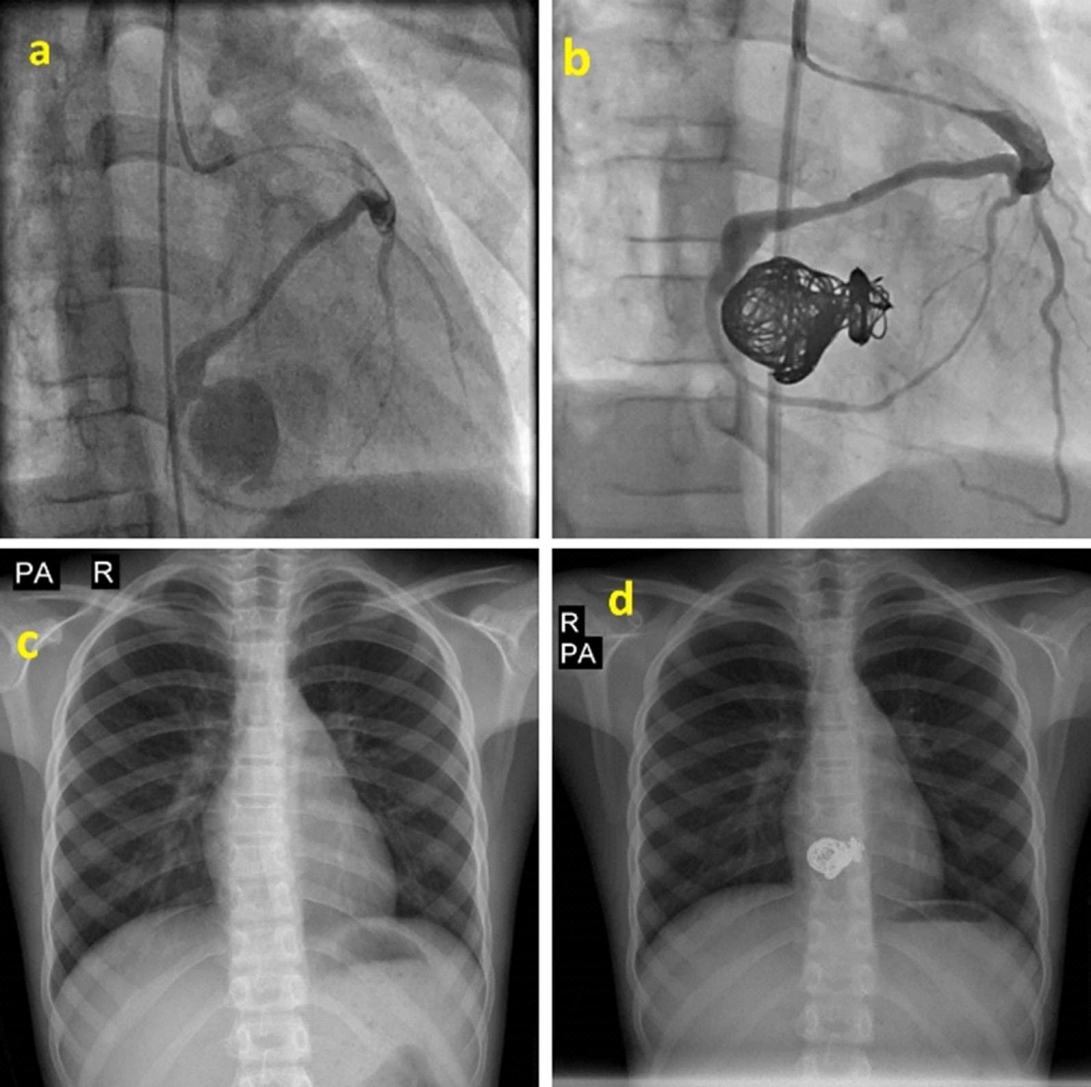
Large coronary aneurysm with RCA to RV before and after intervention. **a**, **b**; Large coronary aneurysm with RCA to RV fistula that closed via retrograde approach with 8 coils. **c**, **d**; Patient's CXR before and after intervention

All of the residual shunt seen in the cases of CAFs were closed by coil, and there is a significant correlation between device coil and residual shunt (*P*-value ≤ 0.05) (Figs. 1–2).

**Fig. 3:**
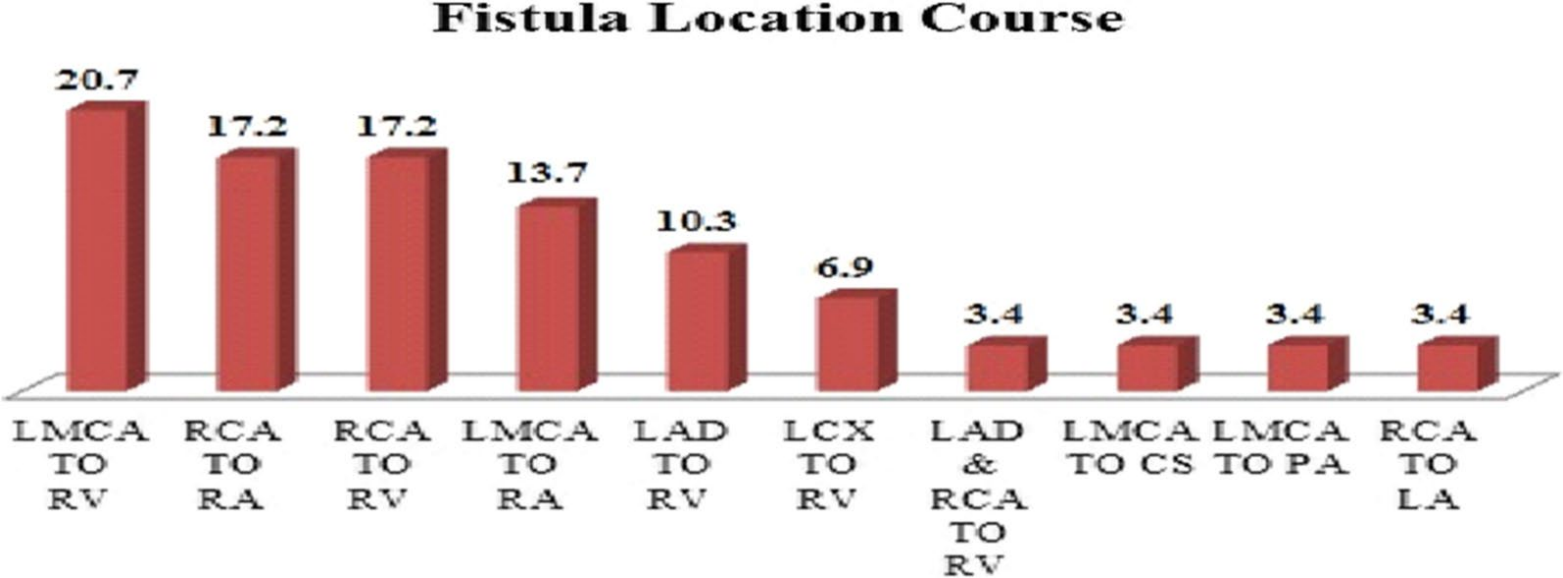
Different coronary artery fistula drainage sites in patients suffering coronary artery fistulas

In terms of delivery system approach, of the 29 patients studied, 31% (9 patients) were treated in an antegrade approach via the aorta and the coronary ostium and 69% (20 patients) were treated via a retrograde approach from the venous side. Of the 7 patients who had minimal residual shunt, 14% (1 patient) was in the antegrade approach group and 86% (6 patients) were in the retrograde approach group. Also, of the 5 patients who had a significant residual shunt, all 5 patients (100%) were in the retrograde approach group. As shown in Table 2, in the study of the relationship between residual shunt and antegrade or retrograde approach, a significant relationship was observed (*p* ≤ 0.05) , which indicated that most of the residual shunts of the patients were in the retrograde approach group. This may well be explained by the fact that the fistula that was treated via a retrograde approach was much larger than those treated by an antegrade approach. Regarding post-intervention events, significant complications occurred in 4 patients: one external iliac artery thrombosis in one patient treated with Alteplase, one case of transient PSVT occurred in a patient with RCA to RV fistula, one case of transient ST-T wave changes was observed in a patient with complex lesion (LAD and RCA to RV fistula), which resolved during follow-up (normalization of the ECG and troponin evaluation) after heparin infusion and one case presented with mild pericardial effusion that resolved spontaneously within the follow-up time. All 4 patients with complications had retrograde approach. It should be noted that coronary artery injury, device dislocation, coronary dissection or ischemia were found in none of the patients. No death was also observed (Fig. 3).

**Table 2:** The association between residual shunt and delivery system approach

Residual shunt	Delivery system approach		Total	*p*-value
	Retrograde	Antegrade		
No residual shunt	9 (31.0%)	8 (27.6%)	17 (58.6%)	0.05
Small residual shunt	6 (20.7%)	1 (3.4%)	7 (24.1%)	
Significant residual shunt	5 (17.2%)	0	5 (17.2%)	
Total	20 (69.0%)	9 (31.0%)	29 (100%)

There was no correlation between age of patients and residual shunt or complications.

## Discussion

Although CAFs are a relatively rare anomaly, they may cause complications such as heart failure, ischemia, thrombosis, arrhythmia, and endocarditis [17]. CAFs’ closure can be surgically performed either by external ligation of the fistula or by internal patching of the orifice; however, the surgical procedure carries the risks of cardiopulmonary bypass and median sternotomy [18]. The first successful treatment of fistula using the trans-catheter method was reported by Reidy et al. in 1983 [19]. Since then, trans-catheter coronary artery fistula closure has evolved in children as the preferred treatment over surgery. Mavroudis et al. [20] recommend device fistula closure in patients who meet the following criteria: absence of multiple fistulas, presence of a single narrow drain, lack of large branching vessels, and secure access to the coronary artery supplying the fistula; in this study, also we excluded CAFs who had complex cardiac lesion. To date, the studies on CAFs repair have shown that although successful closure of the fistula using inflatable balloons, polyvinyl alcohol foam, and umbrellas has been reported, the use of coils is currently considered the best method [21]. This was consistent with our study in which the most frequent fistula closure (79.3%) was performed with the use of coils and so there was a significant correlation between residual shunt and coil occlusion (P-VALUE:0.021). Also, according to studies, due to the improvement of coil occlusion methods, since the risk of this method is low, therefore, there was no report of death in follow-up group [21, 22]. Based on a study conducted by Christmann et al. in 2017 [23], echocardiography is an excellent method for assessing ventricular size, ventricular function, coronary artery size, and fistula leakage after device closure; therefore, we used it for monitoring after closing the fistula. In the pointed study, Christmann et al. [23] stated that the origin of CAFs was identifiable by echocardiography in only 80 patients out of 194 patients (41.2%), of which 77.5% were from the left coronary artery. In our study, the origin of CAF was known by echocardiography and CT-angiography in all 29 patients, of which 58.6% originated in the left coronary artery. Christmann et al. [23] also stated that the fistula drainage site was diagnosed in 157 patients out of 194 patients (80.9%) that in our study the fistula drainage site was identifiable before intervention in all cases. This is because we used CT-angiography for better evaluation in all cases where echocardiography was not efficient. In the above study, the most frequent drainage site was the right side of the heart (right atrium, right ventricle or main pulmonary artery) in 80.6% of the cases, which was consistent with our study. In our study, the drainage site was 93.1% on the right side of the heart, and the most common site of drainage in right side of the heart was right ventricle that was followed by the right atrium, the coronary sinus and then pulmonary artery trunk. Regarding cardiac comorbidities, in the study by Christmann et al. [23], out of 194 patients, 4 had associated congenital anomalies, including 1 patient with ASD_2_, one patient with TOF, one patient with ALCAPA, and one patient with PA + VSD. In our study, there were 5 patients with congenital heart defects including 2 patients with ASD_2_, 1 patient with PFO, 1 patient with a single coronary ostium and 1 patient with TOF who underwent complete surgical correction. There was no correlation between associated anomalies and residual shunt or complications. In the study presented by Zhu et al. [24], trivial-mild residual shunt was reported in 25% of cases, and according to the results of our study, small residual shunt was observed in 24.2% of cases that all closed spontaneously during the follow-up. Also, 17.2% of our cases had a significant residual shunt and all of the above were successfully closed during subsequent catheter intervention. In concordance with the study by Vijay Trehan et al. [25], we did not have any case or CAFs recurrence. In a study by Lourie et al. [26], it was stated that 45% of trans-catheter access cases were performed by an antegrade method and 55% were closed via a retrograde method; in our study 31% of cases were treated by an antegrade approach and 69% of cases were managed by a retrograde approach. Regarding post intervention complications Abdi Jama et al. [27] reported 4 complications in 36 procedure; 1 case of coronary spasm,2 cases of coil embolization and one case of complete occlusion of LAD. In our study, there was also a significant complication in 4 patients (see above) but only one was attributed to the coil placement in the coronary artery. Regarding post-intervention management, heparin infusion was routinely performed for 48 to 72 h, then clopidogrel continued for 1 to 3 months, based on the fistula anatomy, and low-dose aspirin (3–5 mg/kg) was continued until the coronary origin size was normal in transthoracic echocardiogram or CT-angiogram. Also, in our study, unlike the study of McMahon et al. [18] and Shah et al. [28], no cases of permanent coronary artery damage, device dislocation, coronary dissection, coronary ischemia and coronary dilatation were observed. In addition, no mortality was observed during the follow-up in these patients. Also clinical symptoms, like pulmonary hypertension and volume overload were improved during follow-up.

## Conclusions

Because CAF is a very rare disease, individualized treatment strategies are essential in children with the disease. Treatment options for each coronary artery fistula patient are determined by the anatomy of the lesion as well as the experience of the treating center. If a catheter intervention is planned, a specific focus should be on the exact anatomy of the lesion to choose the correct individual device. Interventional management is today the primary treatment of choice as excellent long-term outcome has been demonstrated.

## Data Availability

All relevant raw data will be freely available to any scientists wishing to use them for non-commercial purposes without breaching confidentiality of participants.

## Abbreviations

CAFs: Coronary artery fistulas
LAD: Left anterior descending artery
RCA: Right coronary artery
LMCA: Left main coronary artery
LCX: Left circumflex artery
PACS: Picture archiving communication system
PFO: Patent foramen ovale
ASD2: Atrial septal defect
TFTC: Tetralogy of Fallot total correction
TOF: Tetralogy of Fallot
RA: Right atrium
RV: Right ventricle
RVOT: Right ventricle out let
PSVT: Paroxysmal supra ventricular tachycardia
ECG: Electro cardio gram
CXR: Chest X-ray
